# The Role of End-of-Life Issues in the Design and Reporting of Cancer Clinical Trials: A Structured Literature Review

**DOI:** 10.1371/journal.pone.0136640

**Published:** 2015-09-01

**Authors:** Jan Gaertner, Vera Weingärtner, Stefan Lange, Elke Hausner, Ansgar Gerhardus, Steffen T. Simon, Raymond Voltz, Gerhild Becker, Norbert Schmacke

**Affiliations:** 1 Department of Palliative Care, University Medical Center Freiburg, Freiburg, Germany; 2 Palliative Care Center of Excellence for Baden-Württemberg, Baden-Württemberg, Germany; 3 Department of Palliative Medicine, University Hospital of Cologne, Cologne, Germany; 4 Institute for Quality and Efficiency in Health Care (IQWiG), Cologne, Germany; 5 Institute for Public Health and Nursing Research (IPP), University of Bremen, Bremen, Germany; 6 Health Sciences Bremen, University of Bremen, Bremen, Germany; 7 Center for Integrated Oncology (CIO) Cologne/Bonn, Cologne/Bonn, Germany; 8 Clinical Trials Unit (BMBF 01KN1106), University Hospital of Cologne, Cologne, Germany; University of Stirling, UNITED KINGDOM

## Abstract

**Background:**

Randomized controlled trials (RCTs) are important sources of information on the benefits and harms patients may expect from treatment options. The aim of this structured literature review by the German Institute for Quality and Efficiency in Health Care was to explore whether and how the end-of-life (EoL) situation of patients with advanced cancer is considered in RCTs investigating anti-cancer treatments.

**Methods:**

Our journal pool comprised 19 medical journals, namely five preselected key general medical journals as well as 14 specialist journals (mainly cancer) identified via a scoping search. We systematically searched these journals in MEDLINE to identify RCTs investigating anti-cancer treatments for the following four cancer types: glioblastoma, lung cancer (stage IIIb-IV), malignant melanoma (stage IV), and pancreatic cancer (search via OVID; November 2012). We selected a representative sample of 100 publications, that is, the 25 most recent publications for each cancer type. EoL was defined as a life expectancy of ≤ two years. We assessed the information provided on (1) the descriptions of the terminal stage of the disease, (2) the therapeutic goal (i.e. the intended therapeutic benefit of the intervention studied), (3) the study endpoints assessed, (4) the authors’ concluding appraisal of the intervention’s effects, and (5) the terminology referring to the patients’ EoL situation.

**Results:**

Median survival was ≤ one year for each of the four cancer types. Descriptions of the terminal stage of the disease were ambiguous or lacking in 29/100 publications. One or more therapeutic goals were mentioned in 51/100 publications; these goals were patient-relevant in 38 publications (survival alone: 30/38; health-related quality of life (HRQoL) or HRQoL and survival: 6/38; symptom control or symptom control and survival: 2/38). Primary endpoints included survival (50%), surrogates (44%), and safety (3%). Patient-reported outcomes (PROs) were assessed in 36/100 RCTs. The implications of treatment-related harms for the patients were discussed in 22/100 appraisals. Terminology referring to the patients’ EoL situation (e.g. “terminal”) was scarce, whereas terms suggesting control of the disease (e.g. “cancer control“) were common.

**Conclusions:**

The EoL situation of patients with advanced cancer should be more carefully considered in clinical trials. Although the investigation and robust reporting of PROs is a prerequisite for informed decision-making in healthcare, they are rarely defined as endpoints and HRQoL is rarely mentioned as a therapeutic goal. Suggestions for improving standards for study design and reporting are presented.

## Introduction

Despite major research efforts and the development of novel therapies, the decision-making process in the care of patients with advanced cancer is challenging: many patients cannot be cured and face death within a fairly short period of time. Advanced cancer can therefore still be considered as a paradigmatic disease for the end-of-life (EoL) situation [1]. The UK National Institute for Health and Clinical Excellence (NICE) defines EoL treatments as “indicated for patients with a short life expectancy, normally less than 24 months (…)” [2].

Patients with advanced cancer have a high burden of physical symptoms (e.g. pain, nausea, and breathlessness), as well as psychological and social distress, spiritual challenges, and inadequate information on the benefits and harms of potential treatments [3–7]. Consequently, besides prolongation of life, the most important therapeutic goal (i.e. the intended therapeutic benefit) of any intervention is to improve health-related quality of life (HRQoL) and reduce symptom burden [8, 9]. When caring for these patients, physicians frequently struggle to advise them with regard to available treatments [10]. For this, they rely on evidence from randomized controlled trials (RCTs) [10]. However, it is still unclear whether the EoL situation of patients (i.e. the terminal stage of the disease, the limited life expectancy, and the patients’ needs) is considered in the design and reporting of cancer trials and is thus clear to the readers of journal publications.

The aim of this structured literature review was therefore to explore whether and how the EoL situation of patients with advanced cancer is considered in RCTs investigating anti-cancer treatments. For this purpose, we assessed the following information reported in the publications of the RCTs: (1) the descriptions of the terminal stage of the disease, (2) the therapeutic goal of the intervention studied, (3) the study endpoints assessed, (4) the authors’ concluding appraisal of the intervention’s effects, and (5) the terminology referring to the patients’ EoL situation.

## Methods

The project was initiated by the German Institute for Quality and Efficiency in Health Care (IQWiG). A review protocol is available in German and can be provided by the authors on request. We conducted the project in a systematic manner following the applicable items in the checklist of the Preferred Reporting Items for Systematic Reviews and Meta-Analyses (PRISMA) statement [11].

### Search

We aimed to provide an overview of the current research and publication culture and therefore focused on key medical and specialist journals and recent publications of RCTs. In a first step we established the journal pool for our literature search. To ensure having a pool of key medical journals, we preselected five general medical journals (“the Big Five”) and identified further relevant specialist journals (mainly cancer) in a scoping search (Table 1). In a second step we systematically searched the total pool of 19 eligible journals in MEDLINE via OVID to identify publications of RCTs investigating anti-cancer treatments for the following four cancer types: glioblastoma, lung cancer (stage IIIb-IV), malignant melanoma (stage IV), and pancreatic cancer. The search covered the period from 2003 to November 14, 2012 (the full search strategy, including the MeSH terms and further key words used, is included in S1 Appendix). For lung cancer, the search was restricted to 2010–2012, as far more studies are available on this topic than on the other three cancer types. We did not search further databases, as the journal sample used in our review was fully included in MEDLINE.

**Table 1:** Inclusion criteria.

IC1	**Patients:** Adults with (i) lung cancer ≥ stage IIIb, (ii) malignant melanoma stage IV, (iii) glioblastoma (including anaplastic astrocytoma) or (iv) pancreatic cancer
IC2	**Intervention:** Disease-modifying anti-cancer therapy (i.e. chemo- or targeted therapy, radiation or surgery)
IC3	**Study type:** Randomized controlled trial
IC4	**Language:** English
IC5	**Journals: I. Predefined (“the Big Five”) journals (IF)** ^**a**^ **:** Annals of Internal Medicine (16.1), British Medical Journal (16.3), Journal of the American Medical Association (30), Lancet (39.2), New England Journal of Medicine (54.4); **II. Specialist journals selected after scoping search** ^b^ **(IF):** Annals of Oncology (6.6), British Journal of Cancer (4.8), British Journal of Surgery (5.2), Cancer (4.9), Clinical Oncology (The Royal College of Radiologists) (2.8), European Journal of Cancer (4.8), Gut (13.3), International Journal of Radiation Oncology (4.2), Journal of Clinical Oncology (18.0), Journal of the National Cancer Institute (15.2), Journal of Neuro-Oncology^c^ (2.8), Lancet Oncology (24.7), Lung Cancer (3.7), Neuro-Oncology^c^ (5.3)
IC6	**Publication date:** 2003–2012, lung cancer: 2010–2012
IC7	**Publication type:** Primary publications of RCTs. Secondary analyses or sub-analyses were not included.

a. IFs added as supplementary information after completion of the project. They refer to the years 2013–2014 (except for the Journal of the American Medical Association: 2015). The numbers are rounded to one decimal place.

b. We searched the Cochrane Library for Cochrane reviews and health technology assessment reports on four types of advanced solid cancer: glioblastoma (including anaplastic astrocytoma), lung cancer (stage IIIb-IV), malignant melanoma (stage IV), and pancreatic cancer (see S2 Appendix for list of reviews). To identify the most relevant specialist journals, we screened the lists of the studies included in the 19 reviews and extracted all publications of studies on these four cancer types (n = 157) as well as the names of the journals they were published in. We then assessed journal frequency: If a specific journal was included three or more times in the list of relevant study publications, then it was included in the journal pool.

c. Selected additionally for glioblastoma, as the search yielded an insufficient number of hits.

IC: inclusion criterion, IF: impact factor, RCT: randomized controlled trial.

### Sample size determination

We planned a sample size of 100 (25 x 4) publications of RCTs, both for pragmatic reasons and because we regarded this to be a sufficiently large and representative sample to draw conclusions on our specific research questions. As we aimed to include an equal number of current RCTs per cancer type to allow for better comparability of results, we selected the 25 most recent publications for each type. We considered this approach to be transparent and reproducible.

### Eligibility criteria

Primary publications of RCTs investigating the effect of disease-modifying therapies on adult patients with specific types of advanced, solid cancer and a median survival of ≤ 24 months were eligible for inclusion [2]. The four cancer types considered were glioblastoma (including anaplastic astrocytoma), lung cancer (stage IIIb-IV), malignant melanoma (stage IV), and pancreatic cancer (Table 1). We selected these life-limiting cancers as paradigmatic examples of diseases that show a “reasonably predictable decline in physical health” [12] over a short period of time (Trajectory 1 according to Murray et al. [12]). Articles that reported secondary data or sub-analyses were excluded.

### Selection of studies

Two reviewers (JG, VW) independently screened titles and abstracts of all articles identified by the search strategy and consulted the full text if necessary. Disagreement was resolved by a third reviewer (SL / NS). Of all eligible study publications, we selected the 25 most recent publications for each cancer type for further analysis.

### Data extraction

Data extractions were independently tested on five publications by two reviewers (VW, JG) and the results discussed with two additional reviewers (NS, SL) to ensure that procedures were standardized. Data from all included studies were extracted into an extraction form by one reviewer (VW) and subsequently checked for accuracy by a second reviewer (JG). Discrepancies were resolved by two additional reviewers (NS, SL). Finally, two reviewers who had not been involved in the extraction of data (STS, RV) randomly selected two studies of each cancer type and checked the accuracy of the data.

The full texts of the eligible publications were hand-searched and the following data and information extracted (when available):

- Basic information: first author, journal, year of publication, study aim, sample size, disease, intervention and control arms, and funding.
- Patient characteristics: stage of the disease, functional performance (e. g. Karnofsky Performance Scale (KPS), Eastern Cooperative Oncology Group (ECOG) score), and results for survival measures.
- Introductory statements on the lethality and terminal stage of disease.
- Statements on the therapeutic goal of the intervention, defined as “the therapeutic benefit that the intervention studied was intended to have for the patients”. Typical examples could be prolongation of life (survival) or improvement of patients’ HRQoL or symptoms. This must not be confused with the primary endpoint.
- Study endpoints and outcome measures.
- Data and related statements on results for serious adverse events as well as severe adverse events (grade 3–5 according to the Common Terminology Criteria of Adverse Events, CTCAE), as recommended by the Consolidated Standards of Reporting Trials (CONSORT) guidelines [13, 14].
- Authors’ concluding appraisals of the intervention’s effects (benefits and harms).
- Terminology referring to the patients’ EoL situation, including the classification of available treatment options. The PDFs of the full texts were also electronically screened to double-check for the use of core terminology (see S3 Appendix).

### Analyses

The extracted information was summarized and analyzed descriptively according to the following specific approaches:

*Descriptions of the terminal stage of the disease*: “Unambiguous” was defined as precise information on the prognosis of the disease (e.g. median survival, lethality mentioned). “Ambiguous” included descriptions such as “poor prognosis” or “advanced stage”, which did not explicitly address lethality or provide prognosis data. Missing descriptions were also noted.

*Therapeutic goals*: Survival and patient-reported outcomes (PROs) (i.e. HRQoL) were considered patient-relevant therapeutic goals in this patient sample based on recommendations by the World Health Organization (WHO) and the National Comprehensive Cancer Network (NCCN), as well as on preferences of European citizens determined in a population-based survey [8, 9, 15]. Frequently used terms such as “anti-tumor activity” and “efficacy” were rated as “unclear” if no further details were specified.

*Study endpoints*: These were categorized as:

- survival,
- PROs (i.e. HRQoL),
- safety measures,
- (combined) surrogate measures (e.g. tumor size, laboratory assessments, progression-free survival (PFS)),
- other endpoints.

*Authors’ appraisal of the intervention’s effect*: In accordance with the CONSORT recommendations, we assessed whether the authors provided appraisals of the realistic benefits and harms of the intervention in the discussion [13, 14] and categorized them as follows:

- Intervention is superior to control intervention(s) or associated with relevant benefits.
- Intervention is equivalent (“non-inferior”) to control intervention(s).
- Intervention is inferior to control intervention(s), or futile or harmful.
- Additional studies of the intervention are recommended.

Four researchers (JG, NS, SL, VW) explored whether the authors´ appraisal was substantiated by data provided in the publications according to the CONSORT criteria [13, 14]. For example, a statement such as “the treatment is a valuable therapeutic option since it was well tolerated and resulted in a significantly higher tumor-response rate” required data on adverse events for both groups to claim sufficient substantiation, while a statement in the results section such as “no new safety issues were identified” was rated as unclear. Notably, the *relevance* of the authors’ appraisal was not judged, that is, considering the example above, an improvement in a surrogate measure (“tumor response rate”) would be the rationale for recommending the intervention. Although from the research, clinical, and patient view it is unclear whether the improved response rate would result in an increased survival or a better HRQoL, we categorized such statements as “substantiated” because the effect claimed is supported by data provided in the publication.

## Results

**Fig 1:**
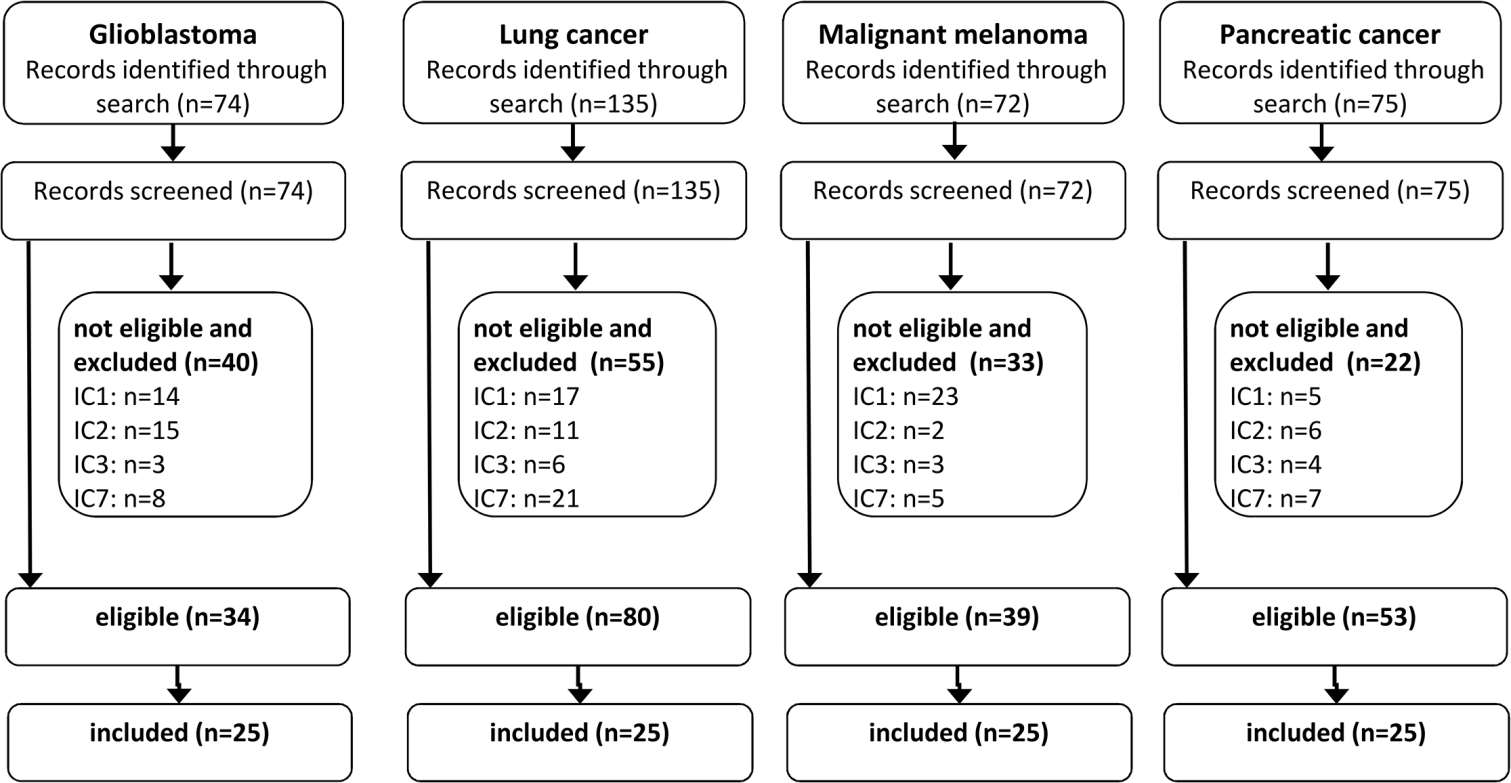
Flowchart of the literature search and screening results.

The search yielded 394 hits and after de-duplication 396 publications were screened (glioblastoma: 74, lung cancer: 135, malignant melanoma: 72, pancreatic cancer: 75). Of these, 206 met the inclusion criteria (34/74, 80/135, 39/72, 53/75), (Fig 1). As planned, the 25 most recent eligible publications per cancer type were analyzed (total n = 100; see S4 Appendix for study details and references).

### Study characteristics

Median survival of patients in the intervention arms varied between 7.5 (range 3.5–23.0) months in pancreatic cancer to 12.3 (4.4–24.6) months in lung cancer. Median survival in the control arms varied between 8.2 (2.3–29.8) months in pancreatic cancer to 11.4 (3.9–23.4) months in lung cancer (see S4 Appendix). In 51/100 studies, a good performance status (ECOG status ≤1 or KPS ≥70) was an inclusion criterion.

### Descriptions of the terminal stage of disease

In 71/100 publications authors provided “unambiguous” descriptions of the terminal stage of the disease (Table 2, examples extracted from [16–21]). Descriptions were “ambiguous” in 10/100 and completely lacking in 19/100 publications, the latter varying from two publications on pancreatic cancer to eight on lung cancer.

**Table 2:** Examples of unambiguous and ambiguous descriptions of the terminal stage of disease.

**Examples of unambiguous descriptions**	**Reference**
“If detected and treated at an early stage, melanoma has a cure rate of approximately 90%.3 In contrast, the prognosis for advanced disease is poor with an average 5-year survival rate of 18% and a median survival of 7.8 months.“	[19]
“The majority of patients with pancreatic cancer are diagnosed in the advanced, unresectable stage, when the primary goals of treatment are survival prolongation and symptom palliation. The impact of systemic treatments in these patients is poor”	[18]
“At the time of diagnosis, approximately half of the patients have metastases, and the median survival time barely exceeds 6 months, whereas approximately one-third of patients diagnosed with locally advanced disease have median survival times ranging between 6 and 9 months. Thus, a small proportion of patients are eligible for surgery, the only curative treatment option, at diagnosis”	[21]
**Examples of ambiguous descriptions**	
“Advanced non-small-cell lung cancer (NSCLC) is an often fatal disease.”	[17]
“Moreover, given their short duration of survival (…)”	[16]
“Despite extensive research, the prognosis of advanced pancreatic cancer remains poor”	[20]

### Therapeutic goals

In 51/100 publications, one or more therapeutic goals of the intervention were explicitly mentioned; these goals were patient-relevant in 38 publications (survival alone: 30/38, HRQoL or HRQoL and survival: 6/38, symptom control or symptom control and survival: 2/38); (Table 3, examples extracted from [22–25]). While a patient-relevant therapeutic goal was mentioned in 13/25 publications on pancreatic cancer, this was only the case in 3/25 publications on malignant melanoma. In 13/100 publications, the therapeutic goals were not clearly patient-relevant (e.g. tumor response) and in 49/100 publications the therapeutic goal was unclear or not mentioned at all.

**Table 3:** Therapeutic goals mentioned in the studies.

	Total n = 100	Glioblastoma (incl. AA) n = 25	Lung cancer n = 25	Malignant melanoma n = 25	Pancreatic cancer n = 25
**Patient-relevant TG** ^**a**^	38	10	12	3	13
Survival alone	30	7	11	3	9
Survival & HRQoL	5	2	-	-	3
Survival & symptom control	1	-	-	-	1
HRQoL alone	1	1	-	-	-
Symptom control alone	1	-	1	-	-
**Other TG** ^**b**^	13	2	3	7	1
**TG unclear** ^**c**^ **or not mentioned**	49	13	10	15	11

a. TG was defined as “the therapeutic benefit that the intervention studied was intended to have for the patients”.

b. Examples: “to assess the use of porphyrin fluorescence in malignant glioma after administration of 5-aminolevulinic acid for improving resection as defined by postoperative MRI, and to analyse the effect of resection on progression free survival, neurological morbidity, and type and frequency of treatment after progression” [25]; “Besides determining tumor response rate to LM/TMZ [lomeguatrib/temozolomide], we aimed to test whether the combination could produce tumor shrinkage in patients progressing on TMZ alone” [23].

c. Examples: “to assess efficacy and tumor delivery of cilengitide in patients with recurrent GBM” [22]; “to define the activity of metronomic chemotherapy with either oral etoposide or temozolomide, combined with bevacizumab” [24].

AA: anaplastic astrocytoma, GBM: glioblastoma, HRQoL: health-related quality of life, MRI: magnetic resonance imaging, TG: therapeutic goal.

### Endpoints

One publication did not define a primary endpoint, and six defined more than one primary endpoint, so that the number of primary endpoints differs from the number of included studies (Table 4; also see S4 Appendix for primary endpoint per study). The most common primary endpoints were survival (53/106) and (combined) surrogate measures (47/106). Safety measures were defined as primary endpoints in three publications. PROs were never assessed as a primary endpoint.

**Table 4:** Primary study endpoints.

	Total^a^ n = 106	Glioblastoma (incl. AA) n = 27	Lung cancer n = 25	Malignant melanoma n = 29	Pancreatic cancer n = 25
**Survival** ^**b**^	53	13	13	8	19
**Surrogate measures** ^**c**^	47	12	12	18	5
**PFS**	28	9	8	8	3
**Response parameters** ^**d**^	13	1	3	8	1
**Tumor control** ^**e**^	5	2	1	2	-
**1-year DFS**	1	-	-	-	1
**Safety measures**	3	1	0	1	1
**PROs**	0	0	0	0	0
**Other** ^**f**^	3	1	0	2	0

The numbers of primary endpoints are presented as reported in the publications.

a. One publication did not define any endpoint as the primary one, and six named more than one primary endpoint. Therefore, the number of endpoints differs from the number of included studies.

b. Included median overall survival (time) and survival rates.

c. Included combined surrogate measures such as PFS.

d. Included best overall response, clinical response, objective response rate, and (tumor) response rate.

e. Included early disease progression rate, time to second progression, time to tumor progression, time to treatment failure, and tumor control rate after 6 months.

f. Included proportion of patients with histologically confirmed malignant glioma on central neuropathological review without residual contrast-enhancing tumor on postoperative MRI, time to CNS metastases (time from randomization to the radiological occurrence of CNS failure), and impact of the addition of GM-CSF to the MPS160/ISA-51 vaccine (maximum change in the frequency of peptide-specific cytotoxic T lymphocytes in peripheral blood from pre-treatment levels (tetramer analysis).

AA: anaplastic astrocytoma, CNS: central nervous system, DFS: disease-free survival, GM-CSF: granulocyte macrophage colony-stimulating factor, MRI: magnetic resonance imaging, PFS: progression-free survival, PROs: patient reported outcomes.

Studies in pancreatic cancer more often assessed overall survival and less often assessed surrogate measures as primary study endpoints compared to the other cancer types.

PROs were assessed as any endpoint in 36 publications and the corresponding results were reported in 31/36 publications (glioblastoma: 10/11, lung cancer: 9/10, malignant melanoma: 0/2, pancreatic cancer: 12/13). Survival was assessed as any endpoint in 98/100 publications.

### Authors’ overall appraisal of the intervention

An overall appraisal of the intervention studied was provided in all 100 publications. Authors of 88/100 publications weighted the benefits and harms of the intervention; 22/88 addressed the meaning of these harms for the patient (patient burden, e.g. due to severe nausea).

Superiority (inferiority) of the intervention was claimed in 34 (39)/100 publications. The intervention and control groups were found to be equivalent in three publications and a need for further research was emphasized in 24 appraisals.

Forty-eight appraisals were fully substantiated by the results provided in the publication. The remaining appraisals were not (28/52) or not fully (17/52) substantiated. In seven publications, claims made in the conclusion were not supported by data in the text. Major reasons for inadequate substantiation included:

1. Study outcomes relied on surrogate measures and disregarded the continuous assessment of patient-centered PROs with valid tools. Therefore, a thorough weighting of the intervention’s effect on the two leading therapeutic goals (i.e. prolongation of life and improvement of HRQoL / symptom control) was not possible. Authors’ conclusions often relied on the unproven assumption that an “anti-cancer effect” or increased PFS is synonymous with increased survival.
2. PRO data were sometimes not reported in the primary publication but published separately later.
3. Reporting of treatment-related harms, such as serious and severe adverse events and burdensome symptoms, was often incomplete, not transparent or ambiguous according to CONSORT [13, 14], as unspecific and subjective wording was used (e.g. “the treatment was generally well tolerated" or "no new / unexpected safety issues occurred”).

### Terminology

No publication mentioned the terms *end-of-life (care)*, *terminal (care)* or *advanced care* (*planning*) (Table 5). Three publications mentioned *palliative care*. More frequently used terms were *cancer/disease/tumor control* (31), *best supportive care* (19), *palliative therapy/palliative treatment* (15), *supportive care* (12), and *salvage therapy/salvage treatment* (11).

**Table 5:** Use and definition of terms screened in the publications.

Term /word stem screened	Total^a^	Glio-blastoma (incl. AA) n = 25	Lung cancer n = 25	Malignant melanoma n = 25	Pancreatic cancer n = 25
**Curative**	**14 (0)**	3	2	2	7
Definition	**2**	1	-	1	-
**Curative related terms**	**13 (0)**	-	2	6	5
Definition	**-**				
***Palliative*** ^**b**^	**4 (0)**	-	-	1	3
Definition	**-**				
***Palliative care***	**3 (1)**	1	1 (1)	-	1
Definition	**-**				
***Palliative therapy / palliative treatment***	**15 (1)**	2 (1)	5	2	6
Definition	**-**				
***Palliation***	**3 (0)**	1	-	1	1
Definition	**-**				
***Best supportive care***	**19 (6)**	1	9 (2)	-	9 (4)
Definition	**2**	-	-	-	2
***Supportive care*** ^***c***^	**12 (1)**	3 (1)	3	5	1
Definition	**4**	1	-	3	-
***Supportive therapy / supportive treatment***	**4 (0)**	2	1	-	1
Definition	**-**				
***Terminal (care)***	**-**	-	-	-	-
***End-of-life (care)***	**-**	-	-	-	-
***Advanced care***	**-**	-	-	-	-
***Near death / dying***	**-**	-	-	-	-
***Salvage therapy / salvage treatment***	**11 (0)**	8	-	2	1
Definition	**1**	1	-	-	-
***Cancer-*, *tumor- or disease control***	**31 (5)**	2 (1)	10 (1)	9	10 (3)
Definition	**20**	1	8	6	5

a. Shows the number of publications that used the term in the full text (in brackets: use in the abstract).

b. Counts only the specific adjective: separately analyzed terms (*palliative care* etc.) were not counted here.

c. Counts only the full term, i.e. not counted if included within “best supportive care”.

AA: anaplastic astrocytoma.

*Cancer/disease/tumor control* usually described a study endpoint defined as measuring stable disease and/or complete or partial responses. *Best supportive care* most often referred to the pharmacologic treatment of symptoms (e.g. nausea, cancer pain), whereas the term *supportive treatment* was used inconsistently. *Salvage therapy* referred to disease-modifying interventions that were used for patients with late-stage disease when previous treatments had failed and no other options were available.

## Discussion

### Summary of main findings

Most patients from the RCTs examined in our review died within one year after study enrollment. However, only half of the authors mentioned a therapeutic goal, and this goal was HRQoL in only six publications. PROs were never assessed as a primary endpoint and only about a third of all RCTs assessed PROs at all. Terminology referring to the patients’ EoL situation was rarely used. Limited reporting of PRO data, as well as of harms, were among the main reasons why some of the authors’ appraisals were not substantiated by the corresponding information provided elsewhere in the publications. In addition, these appraisals were often based on unproven assumptions, for example, the hypothesis that increased “anti-tumor activity” is associated with increased survival or improved HRQoL.

### Interpretation of results

#### Therapeutic goals

Although research has identified prolongation of life and improvement of HRQoL as the most important therapeutic goals for the decision-making process when caring for patients with advanced, life-limiting diseases [1, 8, 9], in our sample a patient-relevant therapeutic goal was only mentioned in every third publication and was almost exclusively prolongation of life. In the majority of publications, the therapeutic goal remained unclear or was not clearly patient-relevant (e.g. anti-tumor activity). Although most of the participants in our study pool had a median life expectancy of less than a year, HRQoL was a neglected therapeutic goal in these RCTs. Notably, this is inconsistent with patients’ priorities determined in a large European cohort study indicating that quality of life is the most preferred therapeutic goal in the case of a fatal and progressive disease [8]. Indisputably, preventing or ameliorating the physical, psychosocial and spiritual suffering of patients and their families dealing with a life-limiting illness is one of the most important goals of care for these patients [8, 9, 26]. As a matter of principle, therapeutic decisions in this EoL context should carefully judge the benefit a patient may expect from possible treatment options [8, 9, 26].

#### Study endpoints

Study outcomes that patients directly experience and care about, that is, survival, functional status, symptoms, and HRQoL, have become increasingly important in the light of patient-centered care [27–29].

In our sample, the primary endpoints were almost exclusively (combined) surrogate measures (e.g. tumor response or PFS) or survival. PROs (including HRQoL) were only measured in about a third of the trials and PRO results were often not reported in the primary publication. These findings are supported by previous research [29–33]. Ghimire et al. assessed outcome measures in phase II and phase III trials in advanced lung cancer based on the ClinicalTrials.gov database [31]. They reported that HRQoL was a secondary endpoint in only 20% of phase II and III trials (phase II: 64/459, 14%; phase III: 54/128, 42%), and that other PROs were rarely used [31]. Another German review found that PROs were assessed as endpoints in 29/123 (24%) RCTs that investigated the effects of chemotherapy on breast cancer patients (primary endpoint in 6/123 RCTs) [29].

These findings indicate that the assessment and reporting of patients’ HRQoL and symptom burden is not yet the norm in RCTs in patients with advanced cancer.

#### Authors’ appraisal of the intervention’s benefits and harms

In 2004, the CONSORT group highlighted the importance of wording when reporting harms in RCTs and explicitly recommends avoiding vague statements such as “the drug was generally well tolerated”, which the group considers to be *poor reporting practice* [13]. Ambiguous wording such as “no unexpected safety issues occurred” was commonly used in our study sample and often not substantiated by the data provided, for example, when serious or severe adverse events or burdensome symptoms such as diarrhea, vomiting or skin reactions occurred. The explicit and unambiguous reporting of harms is crucial, especially for the vulnerable patient population studied, and should follow existing guidelines (e.g. CONSORT) [26, 34, 35].

#### Terminology

While wording addressing the patients EoL situation (e.g. “terminal”) was scarcely used, terms related to “control” of the disease (e.g. tumor control, salvage therapy) were common. Content analysis revealed an understanding of salvage therapy that was limited to patients with exhausted treatment options, and whose overall survival time after salvage therapy was quite short [35–38]. This is in contrast to the literal meaning of “salvage” (to save, to rescue [39]), and it remains unclear whether physicians’ and patients’ expectations toward the realistic goal of care may be misled by this wording.

### Implications for future research and for study authors

To enable informed and shared treatment decisions, authors should provide clear information on the overall therapeutic goal of the intervention studied and the terminal stage of the patients’ disease. This would also facilitate the readers´ critical reflection of the intervention’s impact on the patients`lives.

Without evidence on how patients experience treatment effects, patients, clinicians and other stakeholders do not have enough information to make well-informed decisions [40]. Accordingly, leading health institutions have increasingly stressed that PROs are important outcomes in clinical trials in the field of advanced cancer [29, 40–45]. PROs (particularly HRQoL and symptom burden) should therefore be routinely assessed. Recent progress highlights the increasing importance of this topic and provides recommendations for selecting, incorporating and reporting PROs in clinical research [40–42, 46, 47]. In view of the diversity of available PRO instruments, it may be challenging to select the appropriate PRO measure for the study population, intervention, and disease investigated. Disease-specific core outcome sets for clinical trials are currently being developed by the Core Outcome Measures in Effectiveness Trials (COMET, http://www.comet-initiative.org/) initiative. However, a number of validated, generic and tumor-specific instruments already exist in different languages for the assessment of HRQoL and symptom burden, for example, the European Organization for Research and Treatment of Cancer (EORTC) quality-of-life questionnaire and its modules or the Functional Assessment of Cancer Therapy (FACT) questionnaires, which are currently recommended because of the available evidence supporting their psychometric properties and their past use in cancer clinical research [40, 48, 49]. Moreover, specific databases (e.g. the Patient-Reported Outcome and Quality of life Instruments Database, PROQOLID [50]) provide helpful information on the purpose, characteristics, and sources of many PRO measures. Nevertheless, methodological challenges specific to advanced or terminal diseases must be considered to obtain meaningful PRO data (e.g. conduct of frequent assessments to minimize missing data, without overburdening patients; use of specific methods to account for increased attrition) [40]. PRO assessments should be continued throughout the follow-up phase and should not be stopped (e.g. after a treatment switch) [40]. PRO data as well as adverse events should be reported in the primary publication and should meet existing reporting standards [13, 51].

In publications of RCTs investigating advanced disease, misleading language suggesting “control” of the disease (e.g. salvage therapy) and downplaying treatment-related harms should be avoided [13, 52]. Instead, readers’ understanding of the realistic benefits and harms of the intervention and the expected course of the disease should be increased by more precise wording (e.g. life-prolonging intervention) and transparent reporting of harmful or burdensome treatment effects.

It remains unclear why EoL aspects, particularly the value of PROs and HRQoL, were so often neglected.

### Implications for healthcare stakeholders and medical journals

Regulatory agencies and research funders should ensure that the necessary prerequisites are met to allow investigators to conduct a valid assessment of PROs in (advanced) cancer trials.

Journal reviewers and editors should screen submitted manuscripts of cancer trials more critically for selective or poor reporting of harms to avoid the risk of misinterpretation of study results by readers.

### Limitations of the review

Our review focuses on RCTs investigating patients with advanced and incurable cancer. The main limitation is that we restricted our sample to specific journals and publications: We only searched for RCTs on four cancer types that were included in 19 medical journals indexed in a single bibliographic database. Our sample therefore did not include all relevant publications and our results cannot readily be generalized. However, our journal pool comprised major general medical journals, as well as specialist journals identified in a scoping search and, as mentioned above, we consider the four cancer types chosen to be paradigmatic examples of advanced cancer. In addition, similar studies investigating the same and further types of cancer show comparable results [29, 30, 32]. We thus assume that the results of our sample are representative and by and large provide an accurate picture of the current research and publication culture regarding RCTs in patients with advanced cancer.

Finally, our statements on the 100 RCTs included in our sample are based solely on information provided in the primary publications. It should be noted that comparisons of the completeness of reporting in journal publications and other sources (web-based study registries, publicly available assessments of reimbursement dossiers, and unpublished clinical study reports) have shown that journal publications often inadequately report the methods and results of clinical trials [53–55]. However, this is a different research question. Our focus lay on the question as to how the EoL situation of patients with advanced cancer is considered in RCTs published in key general and specialist medical journals, presumably the most commonly used source of information on clinical trials.

## Conclusion

The EoL situation of patients with advanced cancer should be more carefully considered in clinical trials. Although the investigation and robust reporting of PROs is a prerequisite for informed decision-making in healthcare, they are rarely defined as endpoints and HRQoL is rarely mentioned as a therapeutic goal. In addition, the terminology used is frequently over-optimistic.

### Recommendations

We suggest that study sponsors, investigators, and authors adhere to the following recommendations for the design and reporting of RCTs in patients with advanced cancer in order to improve the interpretability of the study results within the context of terminal disease. We also suggest that journal editors and reviewers of the submitted manuscripts, as well as the readers of study publications (e.g. clinicians or health policy decision-makers), critically assess adherence to these recommendations:

1. The therapeutic goal of the intervention (e.g. survival) should be defined in the protocol and the publication.
2. The intervention’s effect on HRQoL should be measured routinely [40–42, 46, 47].
3. PRO assessments should be continued throughout the follow-up phase [40–42, 46, 47].
4. PRO results should be reported in the primary publication [13, 51].
5. PRO results should be explicitly discussed when appraising the interventions’ effect [13, 51].
6. Harms of the intervention should be carefully assessed, reported, discussed and included in the authors’ appraisal of the intervention, avoiding ambiguous wording [13, 51].
7. The use of terminology suggesting control or cure of the disease should be avoided.

## Supporting Information

S1 Appendix Search Strategy. (DOCX)

S2 Appendix List of reviews identified by the scoping search. (DOCX)

S3 Appendix Search terms used for publication screening and target terms. (DOCX)

S4 Appendix Details of the included publications. (DOCX)

S5 Appendix PRISMA checklist. (DOC)
