# Supplementary material for: The Role of End-of-Life Issues in the Design and Reporting of Cancer Clinical Trials: A Structured Literature Review
Source: PLoS One. 2015 Sep 1;10(9):e0136640. doi: 10.1371/journal.pone.0136640 (PMC4556677; doi:10.1371/journal.pone.0136640)
Supplement: S1 Appendix — (DOCX) [file pone.0136640.s001.docx]

**S1 Appendix: Search Strategy** (RCT filter applied: Wong – High specificity strategy [1])

| **#** | **Searches** |
| --- | --- |
| **1** | Glioblastoma/ |
| **2** | Astrocytoma/ |
| **3** | *Glioma/ |
| **4** | glioblastoma*.ab,ti. |
| **5** | (anaplastic adj1 astrocytom*).ab,ti. |
| **6** | ((high-grade* or malignant*) adj5 glioma*).ab,ti. |
| **7** | or/1-6 |
| **8** | *Lung Neoplasms/ |
| **9** | *Carcinoma, Non-Small-Cell Lung/ |
| **10** | *Carcinoma, Small Cell/ |
| **11** | *Bronchial Neoplasms/ |
| **12** | ((lung* or bronchial* or bronchus*) adj4 (cancer* or carcinoma* or tumor*)).ab,ti. |
| **13** | or/8-12 |
| **14** | exp Melanoma/ |
| **15** | melanom*.ab,ti. |
| **16** | or/14-15 |
| **17** | exp Pancreatic Neoplasms/ |
| **18** | ((carcinoma* or adenocarcinoma* or cancer*) adj5 (pancreas* or pancreatic*)).ab,ti. |
| **19** | or/17-18 |
| **20** | or/7,13,16,19 |
| **21** | (unresectable* or inoperable* or recurrent* or palliat* or metastas* or metastatic* or advanced* or terminal care or terminally ill* or hospice*).ab,ti. |
| **22** | ((supportive* or symptomatic*) adj3 (care* or therapy or treatment*)).ab,ti. |
| **23** | Palliative Care/ |
| **24** | Neoplasm Metastasis/ |
| **25** | Lymphatic Metastasis/ |
| **26** | secondary.fs. |
| **27** | or/21-26 |
| **28** | 20 and 27 |
| **29** | randomized controlled trial.pt. |
| **30** | Randomized controlled trial.ab,ti. |
| **31** | or/29-30 |
| **32** | 28 and 31 |
| **33** | (0028-4793 or 0098-7484 or 0140-6736 or 0959-8138 or 0959-535X or 0003-4819 or 1533-4406 or 1538-3598 or 1474-547X or 1756-1833 or 1468-5833 or 1539-3704 or 0732-183X or 0008-543X or 0007-0920 or 0360-3016 or 0027-8874 or 0923-7534 or 0936-6555 or 0169-5002 or 1470-2045 or 0007-1323 or 0959-8049 or 0017-5749 or 1527-7755 or 1097-0142 or 1532-1827 or 1879-355X or 1460-2105 or 1569-8041 or 1433-2981 or 1872-8332 or 1474-5488 or 1365-2168 or 1879-0852 or 1468-3288 or 0002-9955 or 0099-5355 or 0007-1447 or 0305-7399 or 0167-594X or 1573-7373 or 1522-8517 or 1523-5866).is. |
| **34** | and/32-33 |
| **35** | and/7,31,33 [Glioblastom] |
| **36** | limit 35 to yr=“2003 -Current“ |
| **37** | and/13,27,31,33 [Lungenkrebs] |
| **38** | limit 37 to yr=“2010 -Current“ |
| **39** | and/16,27,31,33 [Melanom] |
| **40** | limit 39 to yr=“2003 -Current“ |
| **41** | and/19,27,31,33 [Pankreaskrebs] |
| **42** | limit 41 to yr=“2003 -Current“ |

**Reference**

1. Wong SS, Wilczynski NL, Haynes RB (2006) Comparison of top-performing search strategies for detecting clinically sound treatment studies and systematic reviews in MEDLINE and EMBASE. Journal of the Medical Library Association : JMLA 94: 451-455.
