# Supplementary material for: The Role of End-of-Life Issues in the Design and Reporting of Cancer Clinical Trials: A Structured Literature Review
Source: PLoS One. 2015 Sep 1;10(9):e0136640. doi: 10.1371/journal.pone.0136640 (PMC4556677; doi:10.1371/journal.pone.0136640)
Supplement: S2 Appendix — (DOCX) [file pone.0136640.s002.docx]

**S2 Appendix**

**References of the Cochrane reviews and HTA reports identified in the scoping search**

Reviews of:

- Glioblastoma [1-5]
- Lung cancer [6-13]
- Malignant melanoma [14-16]
- Pancreatic cancer [17-19]

1. Garside R, Pitt M, Anderson R, Rogers G, Dyer M, et al. (2007) The effectiveness and cost-effectiveness of carmustine implants and temozolomide for the treatment of newly diagnosed high grade glioma: a systematic review and economic evaluation (Brief record). Health Technology Assessment: Health Technology Assessment. pp. 1.

2. Hart MG, Grant R, Garside R, Rogers G, Somerville M, et al. (2008) Temozolomide for High Grade Glioma. CD007415.

3. Hart MG, Grant R, Garside R, Rogers G, Somerville M, et al. (2011) Chemotherapy wafers for high grade glioma. CD007294.

4. Hart MG, Grant R, Metcalfe SE (2000) Biopsy versus resection for high grade glioma. CD002034.

5. Stewart L, Burdett S, Glioma M-aTG (2002) Chemotherapy for high-grade glioma. CD003913.

6. Cardona ZAF, Reveiz L, Ospina EG, Yepes A (2008) Palliative endobronchial brachytherapy for non-small cell lung cancer. CD004284.

7. Fuentes R, Bonfill CX, Expósito HJ (2006) Surgery versus radiosurgery for patients with a solitary brain metastasis from non-small cell lung cancer. CD004840.

8. Lester JF, Macbeth F, Toy E, Coles B (2006) Palliative radiotherapy regimens for non-small cell lung cancer. CD002143.

9. Manser R, Wright G, Hart D, Byrnes G, Campbell D, et al. (2005) Surgery for local and locally advanced non-small cell lung cancer. CD004699.

10. Non-Small Cell Lung Cancer Collaborative Group (2010) Chemotherapy and supportive care versus supportive care alone for advanced non-small cell lung cancer. CD007309.

11. Patel N, Lester JF, Coles B, Macbeth F (2005) Prophylactic cranial irradiation for preventing brain metastases in patients undergoing radical treatment for non-small cell lung cancer. CD005221.

12. Pelayo AM, Gallego RÓ, Bonfill CX, Agra VY (2009) Chemotherapy versus best supportive care for extensive small cell lung cancer. CD001990.

13. Reveiz L, Rueda J-R, Cardona AF (2012) Chemotherapy for brain metastases from small cell lung cancer. CD007464.

14. Health Technology Assessment (2011) Ipilimumab for previously treated unresectable malignant melanoma (Project record). Health Technology Assessment: Health Technology Assessment.

15. Nachtnebel A (2011) Ipilimumab for pre-treated patients with advanced/metastatic melanoma (Structured abstract). Vienna: Ludwig Boltzmann Institut fuer Health Technology Assessment (LBIHTA): Ludwig Boltzmann Institut fuer Health Technology Assessment (LBIHTA).

16. Sasse AD, Sasse EC, Clark LGO, Ulloa L, Clark OAC (2007) Chemoimmunotherapy versus chemotherapy for metastatic malignant melanoma. CD005413.

17. BlueCross BlueShield Association (2009) Off-label use of bevacizumab: advanced adenocarcinoma of the pancreas (Structured abstract). Chicago IL: Blue Cross Blue Shield Association (BCBS): Blue Cross Blue Shield Association (BCBS).

18. Moss AC, Morris E, MacMathuna P (2006) Palliative biliary stents for obstructing pancreatic carcinoma. CD004200.

19. Yip D, Karapetis C, Strickland A, Steer CB, Goldstein D (2009) Chemotherapy and radiotherapy for inoperable advanced pancreatic cancer. CD002093.
