# Supplementary material for: The Role of End-of-Life Issues in the Design and Reporting of Cancer Clinical Trials: A Structured Literature Review
Source: PLoS One. 2015 Sep 1;10(9):e0136640. doi: 10.1371/journal.pone.0136640 (PMC4556677; doi:10.1371/journal.pone.0136640)
Supplement: S3 Appendix — (DOCX) [file pone.0136640.s003.docx]

**S3 Appendix: Search terms used for publication screening and target terms**

| **Target terms** | **Electronic search term** |
| --- | --- |
| curative, cure, curable | cur |
| healing, heal | heal |
| dying people, dying phase | dying |
| end-of-life, end-of-life care | end(-)of |
| patients near death | near death |
| palliative, palliation, palliative care / therapy / treatment | palli |
| supportive, supportive care / therapy / treatment | supporti |
| advance care, advance care planning | advance care |
| terminal disease, terminal care | terminal |
| cancer / tumor / tumour / disease control | control |
| salvage therapy / treatment | salvag |
