# Supplementary material for: The Role of End-of-Life Issues in the Design and Reporting of Cancer Clinical Trials: A Structured Literature Review
Source: PLoS One. 2015 Sep 1;10(9):e0136640. doi: 10.1371/journal.pone.0136640 (PMC4556677; doi:10.1371/journal.pone.0136640)
Supplement: S4 Appendix — (DOCX) [file pone.0136640.s004.docx]

**S4 Appendix: Details of the included publications**

S4 Appendix 1: Details of the included glioblastoma studies page 2

S4 Appendix 2: Details of the included lung cancer studies page 3

S4 Appendix 3: Details of the included melanoma studies page 4

S4 Appendix 4: Details of the included pancreatic cancer studies page 5

References page 6ff

**S4 Appendix 1: Details of the included glioblastoma studies**

| **Study** | **Number of patients^a^** | **Intervention^b^** | **Primary endpoint** | **OS (IG / CG) [months]^c^** |
| --- | --- | --- | --- | --- |
| 1. Bogdahn 2011 [[1](#_ENREF_1)] | 145 | sys | tumor control rate | 12.0, 13.1 / 11.0 |
| 2 Brada 2010 [[2](#_ENREF_2)] | 447 | sys | OS | 7.2 / 6.7 |
| 3. Buckner 2006 [[3](#_ENREF_3)] | 451 | sys+r | OS | 11.9 / 10.4 |
| 4. Clarke 2009 [[4](#_ENREF_4)] | 85 | sys+r | OS | 16.4 / n.a. |
| 5. Dresemann 2010 [[5](#_ENREF_5)] | 240 | sys | PFS | n.a. |
| 6. Friedman 2009 [[6](#_ENREF_6)] | 167 | sys | multiple^d^ | 9.2 / 8.7 |
| 7. Gilbert 2012 [[7](#_ENREF_7)] | 30 | sys+surg | PFS | n.a. |
| 8. Gilbert 2010 [[8](#_ENREF_8)] | 42 | sys | safety | n.a. |
| 9. Grabenbauer 2009 [[9](#_ENREF_9)] | 59 | sys+r | PFS | 14.6 / 15.9 |
| 10. Henriksson 2006 [[10](#_ENREF_10)] | 140 | sys+r | OS | 17.3, 10.3 / 10.6, 12.3 |
| 11. Hildebrand 2008 [[11](#_ENREF_11)] | 193 | sys+r | OS | 27.3 / 23.9 |
| 12. Keime-Guibert 2007 [[12](#_ENREF_12)] | 85 | r | OS | 7.3 / 4.2 |
| 13. Kim 2011 [[13](#_ENREF_13)] | 82 | sys+r | OS | 28.4 / 18.9 |
| 14. Kunwar 2010 [[14](#_ENREF_14)] | 296 | sys+surg | OS | 9.1 / 8.8 |
| 15. Levin 2006 [[15](#_ENREF_15)] | 162 | sys+r+surg | OS | 10.7 / 9.5 |
| 16. Malmström 2012 [[16](#_ENREF_16)] | 291 | sys+r | OS | 8.3 / 7.5, 6.0 |
| 17. Reardon 2011 [[17](#_ENREF_17)] | 23 | sys | PFS | 3.2, 4.8 / n.a. |
| 18. Reardon 2008 [[18](#_ENREF_18)] | 81 | sys | PFS | 1.6, 2.5 / n.a. |
| 19. Sotelo 2006 [[19](#_ENREF_19)] | 30 | sys+r+surg | OS | 24.0 / 11.0 |
| 20. Stummer 2006 [[20](#_ENREF_20)] | 322 | m+s | PFS | n.a. |
| 21. Stupp 2012 [[21](#_ENREF_21)] | 237 | r | OS | 6.6 / 6.0 |
| 22. van den Bent 2009 [[22](#_ENREF_22)] | 110 | sys | PFS | 7.7 / 7.3 |
| 23. Wick 2012 [[23](#_ENREF_23)] | 412 | sys | OS | 8.6 / 9.6 |
| 24. Wick 2010 [[24](#_ENREF_24)] | 226 | sys | PFS | 6.6 / 7.13 |
| 25. Wick 2009 [[25](#_ENREF_25)] | 318 | sys | other^e^ | 72.1 / 82.6 |
| a. Number of randomized patients.  b. Intervention studied.  c. Median overall survival is presented when data were available (data presented as days or weeks were approximated in months by dividing the data by 30 or 4, respectively; data on survival rates not considered here). If more than one arm was studied, multiple numbers are displayed in the related group.  d. PFS and complete or partial response.  e. “Time from operation to treatment failure (stratified for therapy in the intention-to-treat (ITT) population”.  CG: control group, IG: intervention group, n.a.: data not available, OS: median overall survival, PFS: progression-free survival, r: radiotherapy, surg: surgery, sys: systemic therapy (e.g. i.v. or oral chemotherapy, antibody treatment). | | | | |

**S4 Appendix 2: Details of the included lung cancer studies**

| **Study** | **Number of patients^a^** | **Intervention^b^** | **Primary endpoint** | **OS^c^ (IG / CG) [months]** |
| --- | --- | --- | --- | --- |
| Ahn MJ [[26](#_ENREF_26)] | 73 | sys | PFS | n.a. |
| Atagi S [[27](#_ENREF_27)] | 200 | sys+r | OS | 22.4 / 16.9 |
| Ciuleanu T [[28](#_ENREF_28)] | 424 | sys | OS | 5.3 / 5.5 |
| Gridelli C [[29](#_ENREF_29)] | 760 | sys | OS | 8.7 / 11.6 |
| Hoang T [[30](#_ENREF_30)] | 546 | sys | OS | 16.9 / 15.3 |
| Jänne PA [[31](#_ENREF_31)] | 181 | sys | PFS | 24.6 / 19.8 |
| Kim ST [[32](#_ENREF_32)] | 96 | sys | RR | n.a. |
| LeCaer H [[33](#_ENREF_33)] | 94 | sys | TTSP | 4.4 / 3.9 |
| Lee JS [[34](#_ENREF_34)] | 924 | sys | OS | 8.5 / 7.8 |
| Manegold C [[35](#_ENREF_35)] | 839 | sys | OS | 11.0 / 10.7 |
| Miller VA [[36](#_ENREF_36)] | 585 | sys | OS | 10.8 / 12.0 |
| Niho S [[37](#_ENREF_37)] | 180 | sys | PFS | 22.8 / 23.4 |
| Paz-Ares L [[38](#_ENREF_38)] | 939/539 | sys | PFS | n.a. |
| Paz-Ares L [[39](#_ENREF_39)] | 904 | sys | OS | 12.4 / 12.5 |
| Pesce GA [[40](#_ENREF_40)] | 59 | sys+r | OS | 6.3 / 4.9 |
| Ramalingam SS [[41](#_ENREF_41)] | 172 | sys | PFS | 8.1, 12.1 / 8.1 |
| Rosell R [[42](#_ENREF_42)] | 173 | sys | PFS | 19.3 / 19.5 |
| Scagliotti 2012a [[43](#_ENREF_43)] | 1090 | sys | OS | 13.0 / 11.0 |
| Scagliotti 2012b [[44](#_ENREF_44)] | 960 | sys | OS | 9.0 / 8.5 |
| Socinski 2012 [[45](#_ENREF_45)] | 1052 | sys | ORR | 12.1 / 11.2 |
| Soria 2011 [[46](#_ENREF_46)] | 213 | sys | ORR | 9.8, 13.9, 14.3 / 10.1, 15.1 |
| Wang 2012 [[47](#_ENREF_47)] | 65 | sys+r | OS | 20.2 / 13.5 |
| Witta 2012 [[48](#_ENREF_48)] | 132 | sys | PFS | 8.9 / 6.7 |
| Zarogoulidis 2012 [[49](#_ENREF_49)] | 130 | sys | OS | 15.9, 11.6 / 10.0 |
| Zhang, 2012 [[50](#_ENREF_50)] | 296 | sys | PFS | 18.7 / 16.9 |
| a. Number of randomized patients.  b. Intervention studied.  c. Median overall survival is presented when data were available (data presented as days or weeks were approximated in months by dividing the data by 30 or 4, respectively; data on survival rates not considered here). If more than one arm was studied, multiple numbers are displayed in the related group.  CG: control group, IG: intervention group, n.a.: data not available, ORR: complete or partial response, OS: median overall survival, PFS: progression-free survival, r: radiotherapy, RR response rate, sys: systemic therapy (e.g. i.v. or oral chemotherapy, antibody treatment), TTSP time to second progression. | | | | |

**S4 Appendix 3: Details of the included melanoma studies**

| **Study** | **Number of patients^a^** | **Intervention^b^** | **Primary endpoint** | **OS (IG / CG) [months]^c^** |
| --- | --- | --- | --- | --- |
| Atkins 2008 [[51](#_ENREF_51)] | 416 | sys | OS | 9.0 / 8.7 |
| Bedikian 2010 [[52](#_ENREF_52)] | 393 | sys | OS | 8.9 / 7.5 |
| Camacho 2009 [[53](#_ENREF_53)] | 89 | sys | best overall response | 10.0, 11.5 /n.a. |
| Celis 2007 [[54](#_ENREF_54)] | 28 | vaccine | other^d^ | 6.1, 9.1 / 9.1 |
| Chapman 2011 [[55](#_ENREF_55)] | 675 | sys | OS, PFS | n.a. |
| Chairion-Sileni 2011 [[56](#_ENREF_56)] | 149 | sys | other^e^ | 8.4 / 8.7 |
| Eisen 2010 [[57](#_ENREF_57)] | 306 | sys | OS | 5.9 / 7.4 |
| Flaherty 2012 [[58](#_ENREF_58)] | 322 | sys | PFS | n.a. |
| Glaspy 2009 [[59](#_ENREF_59)] | 294 | sys | OS | 6.8, 7.2 / n.a. |
| Hauschild 2012 [[60](#_ENREF_60)] | 250 | sys | PFS | n.a. |
| Hauschild 2009 [[61](#_ENREF_61)] | 270 | sys | PFS | 10.5 / 10.5 |
| Hersey 2010 [[62](#_ENREF_62)] | 112 | sys | multiple^f^ | 12.6, 9.4 /n.a. |
| Hodi 2010 [[63](#_ENREF_63)] | 676 | sys | OS | 10.0 / 10.1 / 6.4 |
| Kim 2012 [[64](#_ENREF_64)] | 214 | sys | PFS | 12.3 / 8.6 |
| Maio 2010 [[65](#_ENREF_65)] | 488 | sys | ORR | 9.3, 8.6, 10.3 / 9.3 / 6.6 |
| McDermott 2007 [[66](#_ENREF_66)] | 101 | sys | PFS | 11.4 / 12.8 |
| O’Day 2011 [[67](#_ENREF_67)] | 129 | sys | PFS | 11.0, 15.0, 9.8 / 8.0 |
| O’Day 2009 [[68](#_ENREF_68)] | 133 | sys | OS | 11.9 / 7.8 |
| Patel 2011 [[69](#_ENREF_69)] | 859 | sys | OS | 9.2 / 9.4 |
| Ranson 2007 [[70](#_ENREF_70)] | 104 | sys | RR | 7.6 / 7.7 |
| Robert 2011 [[71](#_ENREF_71)] | 250 | sys | OS | 11.2 / 9.1 |
| Schwartzentruber 2011 [[72](#_ENREF_72)] | 185 | sys | clinical response^g^ | 17.8 / 11.1 |
| Tarhini 2009 [[73](#_ENREF_73)] | 64 | sys | tumor RR | n.a. |
| Weber 2009 [[74](#_ENREF_74)] | 176 | sys | ORR | 9.4, 8.4, 9.0 / 11.7 |
| Wolchok 2010 [[75](#_ENREF_75)] | 217 | sys | best overall response | 11.4, 8.7, 8.6 |
| a. Number of randomized patients.  b. Intervention studied.  c. Median overall survival is presented when data were available (data presented as days or weeks were approximated in months by dividing the data by 30 or 4, respectively; data on survival rates not considered here). If more than one arm was studied, multiple numbers are displayed in the related group.  d. Impact of the addition of GM-CSF to the MPS160/ISA-51 vaccine (maximum change in the frequency of peptide-specific cytotoxic T lymphocytes in peripheral blood from pretreatment levels.  e. time to CNS metastases  f. Objective tumor response rate, early disease progression rate (proportion of patients with disease progression at the end of two treatment cycles), and time to progression.  g. Modified World Health Organization criteria.  CG: control group, CNS: central nervous system, IG: intervention group, n.a.: data not available, OS: median overall survival, ORR: objective response rate, PFS: progression-free survival, RR response rate; sys: systemic therapy (e.g. i.v. or oral chemotherapy, antibody treatment), TU: tumor. | | | | |

**S4 Appendix 4: Details of the included pancreatic cancer studies**

| **Study** | **Number of patients^a^** | **Intervention^b^** | **Primary endpoint** | **OS (IG / CG) [months]^c^** |
| --- | --- | --- | --- | --- |
| Chauffert 2008 [[76](#_ENREF_76)] | 119 | sys+r | OS | 8.6 / 13.3 |
| Ciuleanu 2009 [[77](#_ENREF_77)] | 303 | sys | OS | 3.4 / 2.8 |
| Colucci 2010 [[78](#_ENREF_78)] | 400 | sys | OS | 7.2 / 8.3 |
| Conroy 2011 [[79](#_ENREF_79)] | 342 | sys | OS, tumor RR | 11.1 / 6.8 |
| Cunningham 2009 [[80](#_ENREF_80)] | 533 | sys | OS | 7.1 / 6.2 |
| Dahan 2012 [[81](#_ENREF_81)] | 202 | sys | OS | 6.7 / 8.0 |
| Kindler 2011 [[82](#_ENREF_82)] | 632 | sys | OS | 8.5 / 8.3 |
| Kindler 2010 [[83](#_ENREF_83)] | 602 | sys | OS | 5.8 / 5.9 |
| Kulke 2009 [[84](#_ENREF_84)] | 259 | sys | OS | 4.5, 3.3, 4.1, 4.0 / n.a. |
| Loeherer 2011 [[85](#_ENREF_85)] | 74 | sys+r | OS | 11.1 / 9.2 |
| Löhr 2012 [[86](#_ENREF_86)] | 212 | sys | n.a. | 8.1, 8.7, 9.3 / 6.8 |
| Meyer 2010 [[87](#_ENREF_87)] | 33 | sys | safety | n.a. |
| Nakai 2012 [[88](#_ENREF_88)] | 106 | sys | PFS | 13.5 / 8.8 |
| Neoptolemos 2010 [[89](#_ENREF_89)] | 1088 | sys+surg | OS | 23.0 / 23.6 |
| Pelzer 2011 [[90](#_ENREF_90)] | 46 | sys | OS | 4.8 / 2.3 |
| Philip 2010 [[91](#_ENREF_91)] | 545 | sys | OS | 6.3 / 5.9 |
| Poplin 2009 [[92](#_ENREF_92)] | 824 | sys | OS | 6.2, 5.7 / 4.9 |
| Raymond 2011 [[93](#_ENREF_93)] | 171 | sys | PFS | n.a. |
| Regine 2008 [[94](#_ENREF_94)] | 538 | sys+r+surg | OS | 20.5 / 16.9 |
| Spano 2008 [[95](#_ENREF_95)] | 103 | sys | OS | 6.9 / 5.6 |
| Van Cutsem 2009 [[96](#_ENREF_96)] | 607 | sys | OS | 7.1 / 6.0 |
| Wilkowski 2009 [[97](#_ENREF_97)] | 95 | sys+r | OS | 9.3. 7.3 / 9.6 |
| Yao 2011 [[98](#_ENREF_98)] | 410 | sys | PFS | n.a. |
| Yoo 2009 [[99](#_ENREF_99)] | 61 | sys | OS | 1.6 / 1.6 |
| Yoshitomi 2008 [[100](#_ENREF_100)] | 100 | sys | 1-year DFS | 21.2 / 29.8 |
| a. Number of randomized patients.  b. Intervention studied.  c. Median overall survival is presented when data were available (data presented as days or weeks were approximated in months by dividing the data by 30 or 4, respectively; data on survival rates not considered here). If more than one arm was studied, multiple numbers are displayed in the related group.  CG: control group, DFS disease-free survival, IG: intervention group, months: months, n.a.: data not available, OS: median overall survival, ORR: complete or partial response, PFS: progression-free survival, r: radiotherapy, RR response rate; surg: surgery, sys: systemic therapy (e.g. i.v. or oral chemotherapy, antibody treatment). | | | | |

**References**

1. Bogdahn U, Hau P, Stockhammer G, Venkataramana NK, Mahapatra AK, et al. (2011) Targeted therapy for high-grade glioma with the TGF-beta2 inhibitor trabedersen: results of a randomized and controlled phase IIb study. Neuro-oncology 13: 132-142.

2. Brada M, Stenning S, Gabe R, Thompson LC, Levy D, et al. (2010) Temozolomide versus procarbazine, lomustine, and vincristine in recurrent high-grade glioma. Journal of Clinical Oncology 28: 4601-4608.

3. Buckner JC, Ballman KV, Michalak JC, Burton GV, Cascino TL, et al. (2006) Phase III trial of carmustine and cisplatin compared with carmustine alone and standard radiation therapy or accelerated radiation therapy in patients with glioblastoma multiforme: North Central Cancer Treatment Group 93-72-52 and Southwest Oncology Group 9503 trials. Journal of Clinical Oncology 24: 3871-3879.

4. Clarke JL, Iwamoto FM, Sul J, Panageas K, Lassman AB, et al. (2009) Randomized phase II trial of chemoradiotherapy followed by either dose-dense or metronomic temozolomide for newly diagnosed glioblastoma. Journal of Clinical Oncology 27: 3861-3867.

5. Dresemann G, Weller M, Rosenthal MA, Wedding U, Wagner W, et al. (2010) Imatinib in combination with hydroxyurea versus hydroxyurea alone as oral therapy in patients with progressive pretreated glioblastoma resistant to standard dose temozolomide. Journal of Neuro-Oncology 96: 393-402.

6. Friedman HS, Prados MD, Wen PY, Mikkelsen T, Schiff D, et al. (2009) Bevacizumab alone and in combination with irinotecan in recurrent glioblastoma. Journal of Clinical Oncology 27: 4733-4740.

7. Gilbert MR, Kuhn J, Lamborn KR, Lieberman F, Wen PY, et al. (2012) Cilengitide in patients with recurrent glioblastoma: the results of NABTC 03-02; a phase II trial with measures of treatment delivery. Journal of Neuro-Oncology 106: 147-153.

8. Gilbert MR, Gonzalez J, Hunter K, Hess K, Giglio P, et al. (2010) A phase I factorial design study of dose-dense temozolomide alone and in combination with thalidomide, isotretinoin, and/or celecoxib as postchemoradiation adjuvant therapy for newly diagnosed glioblastoma. Neuro-oncology 12: 1167-1172.

9. Grabenbauer GG, Gerber KD, Ganslandt O, Richter A, Klautke G, et al. (2009) Effects of concurrent topotecan and radiation on 6-month progression-free survival in the primary treatment of glioblastoma multiforme. International Journal of Radiation Oncology, Biology, Physics 75: 164-169.

10. Henriksson R, Malmström A, Bergström P, Bergh G, Trojanowski T, et al. (2006) High-grade astrocytoma treated concomitantly with estramustine and radiotherapy. Journal of Neuro-Oncology 78: 321-326.

11. Hildebrand J, Gorlia T, Kros JM, Afra D, Frenay M, et al. (2008) Adjuvant dibromodulcitol and BCNU chemotherapy in anaplastic astrocytoma: results of a randomised European Organisation for Research and Treatment of Cancer phase III study (EORTC study 26882). European Journal of Cancer 44: 1210-1216.

12. Keime-Guibert F, Chinot O, Taillandier L, Cartalat-Carel S, Frenay M, et al. (2007) Radiotherapy for glioblastoma in the elderly. New England Journal of Medicine 356: 1527-1535.

13. Kim IH, Park CK, Heo DS, Kim CY, Rhee CH, et al. (2011) Radiotherapy followed by adjuvant temozolomide with or without neoadjuvant ACNU-CDDP chemotherapy in newly diagnosed glioblastomas: a prospective randomized controlled multicenter phase III trial. Journal of Neuro-Oncology 103: 595-602.

14. Kunwar S, Chang S, Westphal M, Vogelbaum M, Sampson J, et al. (2010) Phase III randomized trial of CED of IL13-PE38QQR vs Gliadel wafers for recurrent glioblastoma. Neuro-oncology 12: 871-881.

15. Levin VA, Phuphanich S, Yung WKA, Forsyth PA, Maestro RD, et al. (2006) Randomized, double-blind, placebo-controlled trial of marimastat in glioblastoma multiforme patients following surgery and irradiation. Journal of Neuro-Oncology 78: 295-302.

16. Malmström A, Grønberg BH, Marosi C, Stupp R, Frappaz D, et al. (2012) Temozolomide versus standard 6-week radiotherapy versus hypofractionated radiotherapy in patients older than 60 years with glioblastoma: the Nordic randomised, phase 3 trial. Lancet Oncology 13: 916-926.

17. Reardon DA, Desjardins A, Peters K, Gururangan S, Sampson J, et al. (2011) Phase II study of metronomic chemotherapy with bevacizumab for recurrent glioblastoma after progression on bevacizumab therapy. Journal of Neuro-Oncology 103: 371-379.

18. Reardon DA, Fink KL, Mikkelsen T, Cloughesy TF, O'Neill A, et al. (2008) Randomized phase II study of cilengitide, an integrin-targeting arginine-glycine-aspartic acid peptide, in recurrent glioblastoma multiforme. Journal of Clinical Oncology 26: 5610-5617.

19. Sotelo J, Briceno E, Lopez-Gonzalez MA (2006) Adding chloroquine to conventional treatment for glioblastoma multiforme: a randomized, double-blind, placebo-controlled trial. Annals of Internal Medicine 144: 337-343.

20. Stummer W, Pichlmeier U, Meinel T, Wiestler OD, Zanella F, et al. (2006) Fluorescence-guided surgery with 5-aminolevulinic acid for resection of malignant glioma: a randomised controlled multicentre phase III trial. Lancet Oncology 7: 392-401.

21. Stupp R, Wong ET, Kanner AA, Steinberg D, Engelhard H, et al. (2012) NovoTTF-100A versus physician's choice chemotherapy in recurrent glioblastoma: a randomised phase III trial of a novel treatment modality. European Journal of Cancer 48: 2192-2202.

22. Van den Bent MJ, Brandes AA, Rampling R, Kouwenhoven MCM, Kros JM, et al. (2009) Randomized phase II trial of erlotinib versus temozolomide or carmustine in recurrent glioblastoma: EORTC Brain Tumor Group study 26034. Journal of Clinical Oncology 27: 1268-1274.

23. Wick W, Platten M, Meisner C, Felsberg J, Tabatabai G, et al. (2012) Temozolomide chemotherapy alone versus radiotherapy alone for malignant astrocytoma in the elderly: the NOA-08 randomised, phase 3 trial. Lancet Oncology 13: 707-715.

24. Wick W, Puduvalli VK, Chamberlain MC, Van den Bent MJ, Carpentier AF, et al. (2010) Phase III study of enzastaurin compared with lomustine in the treatment of recurrent intracranial glioblastoma. Journal of Clinical Oncology 28: 1168-1174.

25. Wick W, Hartmann C, Engel C, Stoffels M, Felsberg J, et al. (2009) NOA-04 randomized phase III trial of sequential radiochemotherapy of anaplastic glioma with procarbazine, lomustine, and vincristine or temozolomide. Journal of Clinical Oncology 27: 5874-5880.

26. Ahn MJ, Yang JCH, Liang J, Kang JH, Xiu Q, et al. (2012) Randomized phase II trial of first-line treatment with pemetrexed-cisplatin, followed sequentially by gefitinib or pemetrexed, in East Asian, never-smoker patients with advanced non-small cell lung cancer. Lung Cancer 77: 346-352.

27. Atagi S, Kawahara M, Yokoyama A, Okamoto H, Yamamoto N, et al. (2012) Thoracic radiotherapy with or without daily low-dose carboplatin in elderly patients with non-small-cell lung cancer: a randomised, controlled, phase 3 trial by the Japan Clinical Oncology Group (JCOG0301). Lancet Oncology 13: 671-678.

28. Ciuleanu T, Stelmakh L, Cicenas S, Miliauskas S, Grigorescu AC, et al. (2012) Efficacy and safety of erlotinib versus chemotherapy in second-line treatment of patients with advanced, non-small-cell lung cancer with poor prognosis (TITAN): a randomised multicentre, open-label, phase 3 study. Lancet Oncology 13: 300-308.

29. Gridelli C, Ciardiello F, Gallo C, Feld R, Butts C, et al. (2012) First-line erlotinib followed by second-line cisplatin-gemcitabine chemotherapy in advanced non-small-cell lung cancer: the TORCH randomized trial. Journal of Clinical Oncology 30: 3002-3011.

30. Hoang T, Dahlberg SE, Schiller JH, Mehta MP, Fitzgerald TJ, et al. (2012) Randomized phase III study of thoracic radiation in combination with paclitaxel and carboplatin with or without thalidomide in patients with stage III non-small-cell lung cancer: the ECOG 3598 study. Journal of Clinical Oncology 30: 616-622.

31. Janne PA, Wang X, Socinski MA, Crawford J, Stinchcombe TE, et al. (2012) Randomized phase II trial of erlotinib alone or with carboplatin and paclitaxel in patients who were never or light former smokers with advanced lung adenocarcinoma: CALGB 30406 trial. Journal of Clinical Oncology 30: 2063-2069.

32. Kim ST, Uhm JE, Lee J, Sun JM, Sohn I, et al. (2012) Randomized phase II study of gefitinib versus erlotinib in patients with advanced non-small cell lung cancer who failed previous chemotherapy. Lung Cancer 75: 82-88.

33. LeCaer H, Greillier L, Corre R, Jullian H, Crequit J, et al. (2012) A multicenter phase II randomized trial of gemcitabine followed by erlotinib at progression, versus the reverse sequence, in vulnerable elderly patients with advanced non small-cell lung cancer selected with a comprehensive geriatric assessment (the GFPC 0505 study). Lung Cancer 77: 97-103.

34. Lee JS, Hirsh V, Park K, Qin S, Blajman CR, et al. (2012) Vandetanib versus placebo in patients with advanced non-small-cell lung cancer after prior therapy with an epidermal growth factor receptor tyrosine kinase inhibitor: a randomized, double-blind phase III trial (ZEPHYR). Journal of Clinical Oncology 30: 1114-1121.

35. Manegold C, Van Zandwijk N, Szczesna A, Zatloukal P, Au JSK, et al. (2012) A phase III randomized study of gemcitabine and cisplatin with or without PF-3512676 (TLR9 agonist) as first-line treatment of advanced non-small-cell lung cancer. Annals of Oncology 23: 72-77.

36. Miller VA, Hirsh V, Cadranel J, Chen YM, Park K, et al. (2012) Afatinib versus placebo for patients with advanced, metastatic non-small-cell lung cancer after failure of erlotinib, gefitinib, or both, and one or two lines of chemotherapy (LUX-Lung 1): a phase 2b/3 randomised trial. Lancet Oncology 13: 528-538.

37. Niho S, Kunitoh H, Nokihara H, Horai T, Ichinose Y, et al. (2012) Randomized phase II study of first-line carboplatin-paclitaxel with or without bevacizumab in Japanese patients with advanced non-squamous non-small-cell lung cancer. Lung Cancer 76: 362-367.

38. Paz-Ares L, De Marinis F, Dediu M, Thomas M, Pujol JL, et al. (2012) Maintenance therapy with pemetrexed plus best supportive care versus placebo plus best supportive care after induction therapy with pemetrexed plus cisplatin for advanced non-squamous non-small-cell lung cancer (PARAMOUNT): a double-blind, phase 3, randomised controlled trial. Lancet Oncology 13: 247-255.

39. Paz-Ares LG, Biesma B, Heigener D, Von Pawel J, Eisen T, et al. (2012) Phase III, randomized, double-blind, placebo-controlled trial of gemcitabine/cisplatin alone or with sorafenib for the first-line treatment of advanced, nonsquamous non-small-cell lung cancer. Journal of Clinical Oncology 30: 3084-3092.

40. Pesce GA, Klingbiel D, Ribi K, Zouhair A, Von Moos R, et al. (2012) Outcome, quality of life and cognitive function of patients with brain metastases from non-small cell lung cancer treated with whole brain radiotherapy combined with gefitinib or temozolomide: a randomised phase II trial of the Swiss Group for Clinical Cancer Research (SAKK 70/03). European Journal of Cancer 48: 377-384.

41. Ramalingam SS, Spigel DR, Chen D, Steins MB, Engelman JA, et al. (2011) Randomized phase II study of erlotinib in combination with placebo or R1507, a monoclonal antibody to insulin-like growth factor-1 receptor, for advanced-stage non-small-cell lung cancer. Journal of Clinical Oncology 29: 4574-4580.

42. Rosell R, Carcereny E, Gervais R, Vergnenegre A, Massuti B, et al. (2012) Erlotinib versus standard chemotherapy as first-line treatment for European patients with advanced EGFR mutation-positive non-small-cell lung cancer (EURTAC): a multicentre, open-label, randomised phase 3 trial. Lancet Oncology 13: 239-246.

43. Scagliotti GV, Vynnychenko I, Park K, Ichinose Y, Kubota K, et al. (2012) International, randomized, placebo-controlled, double-blind phase III study of motesanib plus carboplatin/paclitaxel in patients with advanced nonsquamous non-small-cell lung cancer: MONET1. Journal of Clinical Oncology 30: 2829-2836.

44. Scagliotti GV, Krzakowski M, Szczesna A, Strausz J, Makhson A, et al. (2012) Sunitinib plus erlotinib versus placebo plus erlotinib in patients with previously treated advanced non-small-cell lung cancer: a phase III trial. Journal of Clinical Oncology 30: 2070-2078.

45. Socinski MA, Bondarenko I, Karaseva NA, Makhson AM, Vynnychenko I, et al. (2012) Weekly nab-paclitaxel in combination with carboplatin versus solvent-based paclitaxel plus carboplatin as first-line therapy in patients with advanced non-small-cell lung cancer: final results of a phase III trial. Journal of Clinical Oncology 30: 2055-2062.

46. Soria JC, Mark Z, Zatloukal P, Szima B, Albert I, et al. (2011) Randomized phase II study of dulanermin in combination with paclitaxel, carboplatin, and bevacizumab in advanced non-small-cell lung cancer. Journal of Clinical Oncology 29: 4442-4451.

47. Wang L, Wu S, Ou G, Bi N, Li W, et al. (2012) Randomized phase II study of concurrent cisplatin/etoposide or paclitaxel/carboplatin and thoracic radiotherapy in patients with stage III non-small cell lung cancer. Lung Cancer 77: 89-96.

48. Witta SE, Jotte RM, Konduri K, Neubauer MA, Spira AI, et al. (2012) Randomized phase II trial of erlotinib with and without entinostat in patients with advanced non-small-cell lung cancer who progressed on prior chemotherapy. Journal of Clinical Oncology 30: 2248-2255.

49. Zarogoulidis K, Eleftheriadou E, Kontakiotis T, Gerasimou G, Zarogoulidis P, et al. (2012) Long acting somatostatin analogues in combination to antineoplastic agents in the treatment of small cell lung cancer patients. Lung Cancer 76: 84-88.

50. Zhang L, Ma S, Song X, Han B, Cheng Y, et al. (2012) Gefitinib versus placebo as maintenance therapy in patients with locally advanced or metastatic non-small-cell lung cancer (INFORM; C-TONG 0804): a multicentre, double-blind randomised phase 3 trial. Lancet Oncology 13: 466-475.

51. Atkins MB, Hsu J, Lee S, Cohen GI, Flaherty LE, et al. (2008) Phase III trial comparing concurrent biochemotherapy with cisplatin, vinblastine, dacarbazine, interleukin-2, and interferon alfa-2b with cisplatin, vinblastine, and dacarbazine alone in patients with metastatic malignant melanoma (E3695): a trial coordinated by the Eastern Cooperative Oncology Group. Journal of Clinical Oncology 26: 5748-5754.

52. Bedikian AY, DeConti RC, Conry R, Agarwala S, Papadopoulos N, et al. (2011) Phase 3 study of docosahexaenoic acid-paclitaxel versus dacarbazine in patients with metastatic malignant melanoma. Annals of Oncology 22: 787-793.

53. Camacho LH, Antonia S, Sosman J, Kirkwood JM, Gajewski TF, et al. (2009) Phase I/II trial of tremelimumab in patients with metastatic melanoma. Journal of Clinical Oncology 27: 1075-1081.

54. Celis E (2007) Overlapping human leukocyte antigen class I/II binding peptide vaccine for the treatment of patients with stage IV melanoma: evidence of systemic immune dysfunction. Cancer 110: 203-214.

55. Chapman PB, Hauschild A, Robert C, Haanen JB, Ascierto P, et al. (2011) Improved survival with vemurafenib in melanoma with BRAF V600E mutation. New England Journal of Medicine 364: 2507-2516.

56. Chiarion-Sileni V, Guida M, Ridolfi L, Romanini A, Del Bianco P, et al. (2011) Central nervous system failure in melanoma patients: results of a randomised, multicentre phase 3 study of temozolomide- and dacarbazine- based regimens. British Journal of Cancer 104: 1816-1821.

57. Eisen T, Trefzer U, Hamilton A, Hersey P, Millward M, et al. (2010) Results of a multicenter, randomized, double-blind phase 2/3 study of lenalidomide in the treatment of pretreated relapsed or refractory metastatic malignant melanoma. Cancer 116: 146-154.

58. Flaherty KT, Robert C, Hersey P, Nathan P, Garbe C, et al. (2012) Improved survival with MEK inhibition in BRAF-mutated melanoma. New England Journal of Medicine 367: 107-114.

59. Glaspy J, Atkins MB, Richards JM, Agarwala SS, O'Day S, et al. (2009) Results of a multicenter, randomized, double-blind, dose-evaluating phase 2/3 study of lenalidomide in the treatment of metastatic malignant melanoma. Cancer 115: 5228-5236.

60. Hauschild A, Grob JJ, Demidov LV, Jouary T, Gutzmer R, et al. (2012) Dabrafenib in BRAF-mutated metastatic melanoma: a multicentre, open-label, phase 3 randomised controlled trial. Lancet 380: 358-365.

61. Hauschild A, Agarwala SS, Trefzer U, Hogg D, Robert C, et al. (2009) Results of a phase III, randomized, placebo-controlled study of sorafenib in combination with carboplatin and paclitaxel as second-line treatment in patients with unresectable stage III or stage IV melanoma. Journal of Clinical Oncology 27: 2823-2830.

62. Hersey P, Sosman J, O'Day S, Richards J, Bedikian A, et al. (2010) A randomized phase 2 study of etaracizumab, a monoclonal antibody against integrin α_v_β_3_, ± dacarbazine in patients with stage IV metastatic melanoma. Cancer 116: 1526-1534.

63. Hodi FS, O'Day SJ, McDermott DF, Weber RW, Sosman JA, et al. (2010) Improved survival with ipilimumab in patients with metastatic melanoma. New England Journal of Medicine 363: 711-723.

64. Kim KB, Sosman JA, Fruehauf JP, Linette GP, Markovic SN, et al. (2012) BEAM: a randomized phase II study evaluating the activity of bevacizumab in combination with carboplatin plus paclitaxel in patients with previously untreated advanced melanoma. Journal of Clinical Oncology 30: 34-41.

65. Maio M, Mackiewicz A, Testori A, Trefzer U, Ferraresi V, et al. (2010) Large randomized study of thymosin α 1, interferon alfa, or both in combination with dacarbazine in patients with metastatic melanoma. Journal of Clinical Oncology 28: 1780-1787.

66. McDermott DF, Sosman JA, Gonzalez R, Hodi FS, Linette GP, et al. (2008) Double-blind randomized phase II study of the combination of sorafenib and dacarbazine in patients with advanced melanoma: a report from the 11715 study group. Journal of Clinical Oncology 26: 2178-2185.

67. O'Day S, Pavlick A, Loquai C, Lawson D, Gutzmer R, et al. (2011) A randomised, phase II study of intetumumab, an anti-α_v_-integrin mAb, alone and with dacarbazine in stage IV melanoma. British Journal of Cancer 105: 346-352.

68. O'Day S, Gonzalez R, Lawson D, Weber R, Hutchins L, et al. (2009) Phase II, randomized, controlled, double-blinded trial of weekly elesclomol plus paclitaxel versus paclitaxel alone for stage IV metastatic melanoma. Journal of Clinical Oncology 27: 5452-5458.

69. Patel PM, Suciu S, Mortier L, Kruit WH, Robert C, et al. (2011) Extended schedule, escalated dose temozolomide versus dacarbazine in stage IV melanoma: final results of a randomised phase III study (EORTC 18032). European Journal of Cancer 47: 1476-1483.

70. Ranson M, Hersey P, Thompson D, Beith J, McArthur GA, et al. (2007) Randomized trial of the combination of lomeguatrib and temozolomide compared with temozolomide alone in chemotherapy naive patients with metastatic cutaneous melanoma. Journal of Clinical Oncology 25: 2540-2545.

71. Robert C, Thomas L, Bondarenko I, O'Day S, Weber J, et al. (2011) Ipilimumab plus dacarbazine for previously untreated metastatic melanoma. New England Journal of Medicine 364: 2517-2526.

72. Schwartzentruber DJ, Lawson DH, Richards JM, Conry RM, Miller DM, et al. (2011) Gp100 peptide vaccine and interleukin-2 in patients with advanced melanoma. New England Journal of Medicine 364: 2119-2127.

73. Tarhini AA, Millward M, Mainwaring P, Kefford R, Logan T, et al. (2009) A phase 2, randomized study of SB-485232, rhIL-18, in patients with previously untreated metastatic melanoma. Cancer 115: 859-868.

74. Weber JS, Zarour H, Redman B, Trefzer U, O'Day S, et al. (2009) Randomized phase 2/3 trial of CpG oligodeoxynucleotide PF-3512676 alone or with dacarbazine for patients with unresectable stage III and IV melanoma. Cancer 115: 3944-3954.

75. Wolchok JD, Neyns B, Linette G, Negrier S, Lutzky J, et al. (2010) Ipilimumab monotherapy in patients with pretreated advanced melanoma: a randomised, double-blind, multicentre, phase 2, dose-ranging study. Lancet Oncology 11: 155-164.

76. Chauffert B, Mornex F, Bonnetain F, Rougier P, Mariette C, et al. (2008) Phase III trial comparing intensive induction chemoradiotherapy (60 Gy, infusional 5-FU and intermittent cisplatin) followed by maintenance gemcitabine with gemcitabine alone for locally advanced unresectable pancreatic cancer: definitive results of the 2000-01 FFCD/SFRO study. Annals of Oncology 19: 1592-1599.

77. Ciuleanu TE, Pavlovsky AV, Bodoky G, Garin AM, Langmuir VK, et al. (2009) A randomised Phase III trial of glufosfamide compared with best supportive care in metastatic pancreatic adenocarcinoma previously treated with gemcitabine. European Journal of Cancer 45: 1589-1596.

78. Colucci G, Labianca R, Di Costanzo F, Gebbia V, Carteni G, et al. (2010) Randomized phase III trial of gemcitabine plus cisplatin compared with single-agent gemcitabine as first-line treatment of patients with advanced pancreatic cancer: the GIP-1 study. Journal of Clinical Oncology 28: 1645-1651.

79. Conroy T, Desseigne F, Ychou M, Bouche O, Guimbaud R, et al. (2011) FOLFIRINOX versus gemcitabine for metastatic pancreatic cancer. New England Journal of Medicine 364: 1817-1825.

80. Cunningham D, Chau I, Stocken DD, Valle JW, Smith D, et al. (2009) Phase III randomized comparison of gemcitabine versus gemcitabine plus capecitabine in patients with advanced pancreatic cancer. Journal of Clinical Oncology 27: 5513-5518.

81. Dahan L, Bonnetain F, Ychou M, Mitry E, Gasmi M, et al. (2010) Combination 5-fluorouracil, folinic acid and cisplatin (LV5FU2-CDDP) followed by gemcitabine or the reverse sequence in metastatic pancreatic cancer: final results of a randomised strategic phase III trial (FFCD 0301). Gut 59: 1527-1534.

82. Kindler HL, Ioka T, Richel DJ, Bennouna J, Letourneau R, et al. (2011) Axitinib plus gemcitabine versus placebo plus gemcitabine in patients with advanced pancreatic adenocarcinoma: a double-blind randomised phase 3 study. Lancet Oncology 12: 256-262.

83. Kindler HL, Niedzwiecki D, Hollis D, Sutherland S, Schrag D, et al. (2010) Gemcitabine plus bevacizumab compared with gemcitabine plus placebo in patients with advanced pancreatic cancer: phase III trial of the Cancer and Leukemia Group B (CALGB 80303). Journal of Clinical Oncology 28: 3617-3622.

84. Kulke MH, Tempero MA, Niedzwiecki D, Hollis DR, Kindler HL, et al. (2009) Randomized phase II study of gemcitabine administered at a fixed dose rate or in combination with cisplatin, docetaxel, or irinotecan in patients with metastatic pancreatic cancer: CALGB 89904. Journal of Clinical Oncology 27: 5506-5512.

85. Loehrer PJ Sr, Feng Y, Cardenes H, Wagner L, Brell JM, et al. (2011) Gemcitabine alone versus gemcitabine plus radiotherapy in patients with locally advanced pancreatic cancer: an Eastern Cooperative Oncology Group trial. Journal of Clinical Oncology 29: 4105-4112.

86. Lohr JM, Haas SL, Bechstein WO, Bodoky G, Cwiertka K, et al. (2012) Cationic liposomal paclitaxel plus gemcitabine or gemcitabine alone in patients with advanced pancreatic cancer: a randomized controlled phase II trial. Annals of Oncology 23: 1214-1222.

87. Meyer T, Caplin ME, Palmer DH, Valle JW, Larvin M, et al. (2010) A phase Ib/IIa trial to evaluate the CCK2 receptor antagonist Z-360 in combination with gemcitabine in patients with advanced pancreatic cancer. European Journal of Cancer 46: 526-533.

88. Nakai Y, Isayama H, Sasaki T, Sasahira N, Tsujino T, et al. (2012) A multicentre randomised phase II trial of gemcitabine alone vs gemcitabine and S-1 combination therapy in advanced pancreatic cancer: GEMSAP study. British Journal of Cancer 106: 1934-1939.

89. Neoptolemos JP, Stocken DD, Bassi C, Ghaneh P, Cunningham D, et al. (2010) Adjuvant chemotherapy with fluorouracil plus folinic acid vs gemcitabine following pancreatic cancer resection: a randomized controlled trial. JAMA 304: 1073-1081.

90. Pelzer U, Schwaner I, Stieler J, Adler M, Seraphin J, et al. (2011) Best supportive care (BSC) versus oxaliplatin, folinic acid and 5-fluorouracil (OFF) plus BSC in patients for second-line advanced pancreatic cancer: a phase III-study from the German CONKO-study group. European Journal of Cancer 47: 1676-1681.

91. Philip PA, Benedetti J, Corless CL, Wong R, O'Reilly EM, et al. (2010) Phase III study comparing gemcitabine plus cetuximab versus gemcitabine in patients with advanced pancreatic adenocarcinoma: Southwest Oncology Group-directed intergroup trial S0205. Journal of Clinical Oncology 28: 3605-3610.

92. Poplin E, Feng Y, Berlin J, Rothenberg ML, Hochster H, et al. (2009) Phase III, randomized study of gemcitabine and oxaliplatin versus gemcitabine (fixed-dose rate infusion) compared with gemcitabine (30-minute infusion) in patients with pancreatic carcinoma E6201: a trial of the Eastern Cooperative Oncology Group. Journal of Clinical Oncology 27: 3778-3785.

93. Raymond E, Dahan L, Raoul JL, Bang YJ, Borbath I, et al. (2011) Sunitinib malate for the treatment of pancreatic neuroendocrine tumors. New England Journal of Medicine 364: 501-513.

94. Regine WF, Winter KA, Abrams RA, Safran H, Hoffman JP, et al. (2008) Fluorouracil vs gemcitabine chemotherapy before and after fluorouracil-based chemoradiation following resection of pancreatic adenocarcinoma: a randomized controlled trial. JAMA 299: 1019-1026.

95. Spano JP, Chodkiewicz C, Maurel J, Wong R, Wasan H, et al. (2008) Efficacy of gemcitabine plus axitinib compared with gemcitabine alone in patients with advanced pancreatic cancer: an open-label randomised phase II study. Lancet 371: 2101-2108.

96. Van Cutsem E, Vervenne WL, Bennouna J, Humblet Y, Gill S, et al. (2009) Phase III trial of bevacizumab in combination with gemcitabine and erlotinib in patients with metastatic pancreatic cancer. Journal of Clinical Oncology 27: 2231-2237.

97. Wilkowski R, Boeck S, Ostermaier S, Sauer R, Herbst M, et al. (2009) Chemoradiotherapy with concurrent gemcitabine and cisplatin with or without sequential chemotherapy with gemcitabine/cisplatin vs chemoradiotherapy with concurrent 5-fluorouracil in patients with locally advanced pancreatic cancer: a multi-centre randomised phase II study. British Journal of Cancer 101: 1853-1859.

98. Yao JC, Shah MH, Ito T, Bohas CL, Wolin EM, et al. (2011) Everolimus for advanced pancreatic neuroendocrine tumors. New England Journal of Medicine 364: 514-523.

99. Yoo C, Hwang JY, Kim JE, Kim TW, Lee JS, et al. (2009) A randomised phase II study of modified FOLFIRI.3 vs modified FOLFOX as second-line therapy in patients with gemcitabine-refractory advanced pancreatic cancer. British Journal of Cancer 101: 1658-1663.

100. Yoshitomi H, Togawa A, Kimura F, Ito H, Shimizu H, et al. (2008) A randomized phase II trial of adjuvant chemotherapy with uracil/tegafur and gemcitabine versus gemcitabine alone in patients with resected pancreatic cancer. Cancer 113: 2448-2456.
